# Supplementary material for: Functional Interaction of Hypoxia-Inducible Factor 2-Alpha and Autophagy Mediates Drug Resistance in Colon Cancer Cells
Source: Cancers (Basel). 2019 May 30;11(6):755. doi: 10.3390/cancers11060755 (PMC6627604; doi:10.3390/cancers11060755)
Supplement: Supplementary file 1 [file cancers-11-00755-s001.pdf]

Supplementary Materials: Functional Interaction of Hypoxia-Inducible Factor 2-Alpha and Autophagy Mediates Drug Resistance in Colon Cancer Cells

Abril Saint-Martin, Jacobo Martínez-Ríos, M. Cristina Castañeda-Patlán, Miguel Angel Sarabia-Sánchez, Nydia Tejeda-Muñoz, Alberto Chinney-Herrera, Gloria Soldevila, Roberto Benelli, Paula Santoyo-Ramos, Alessandro Poggi and Martha Robles-Flores

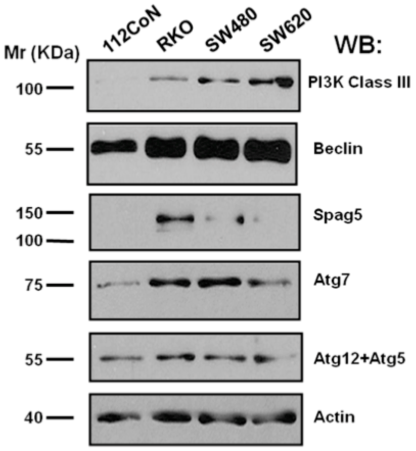

Figure S1. The expression of several autophagy marker proteins was analyzed in colon cell lines by Western blot. The figure shows increased expression of autophagy regulator Atg7, Beclin, Spag5 and PI3K-Class III autophagy markers only in colon cancer cells (RKO, SW480 and SW620). Actin antibody was used to control for equal loading. The images are representative of three independent experiments.

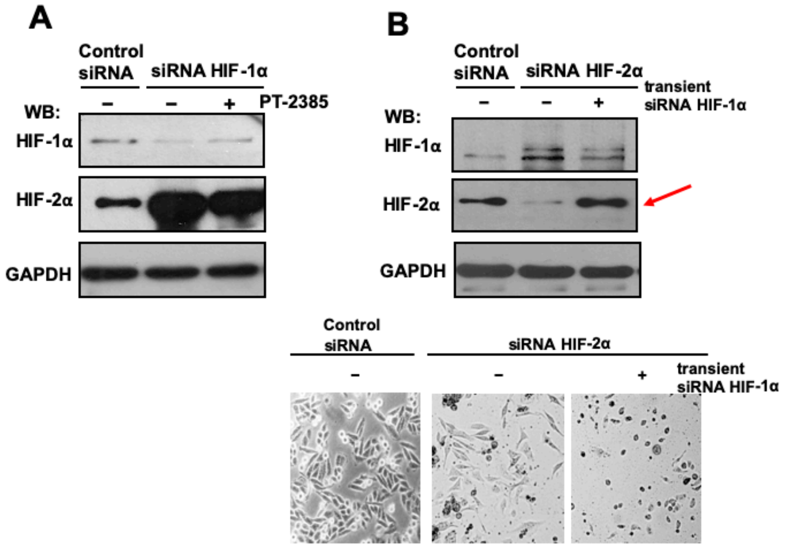

Figure S2. SW480 cells were stable transfected with the control scrambled shRNA plasmid, with HIF-1α RNAi (panel **A**), or with HIF-2α-RNAi (panel **B**). Stable HIF-2α—silenced SW480 cells (panel **B**) were also transiently transfected with HIF-1α-RNAi (third column). In panel **A**, stable HIF-1α-silenced cells were used to incubate in the absence or presence of the HIF-2α inhibitor PT-2385 to mimic a double knockdown effect. HIF-1α or HIF-2α expression analysis was performed by Western blotting analysis and pictures of cells after 36 h post-transfection were taken (40×) and showed in panel **B**. GAPDH was used as loading control. Results shown are representative of three independent experiments using different cell preparations.

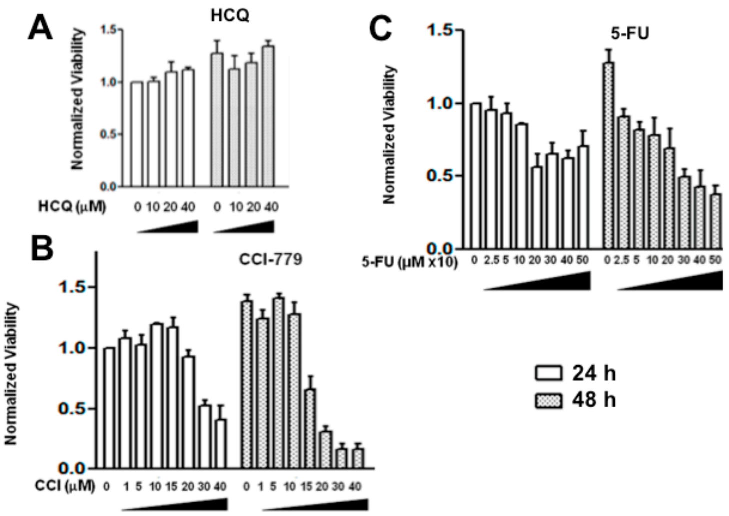

Figure S3. The mTOR inhibitor CCI-779 and the chemotherapeutic drug 5-FU decrease cell viability in a dose- and time-dependent manner, but the autophagy flux inhibitor HCQ did not show cytotoxicity at any dose tested. SW480 cells were grown in the absence or presence of increasing doses of HCQ (**A**), CC1-779 (**B**) or 5-FU alone (**C**), as indicated in the Figure, during 24 or 48 h. The cell viability was examined by the MTT assay at the end of each incubation period, and normalized with respect to that obtained in untreated cells. The bar graph shows the means ± SEM from at least three independent experiments.

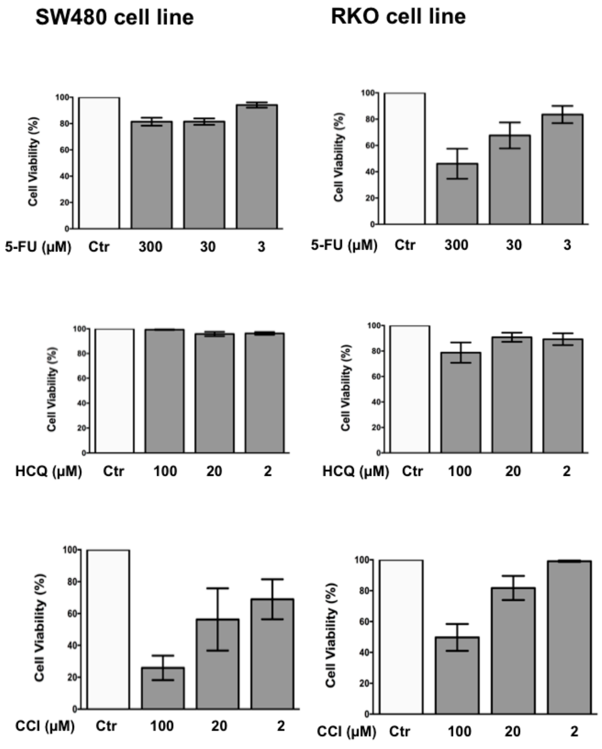

Figure S4. SW480 or RKO cells were grown in the absence (Ctr) or presence of different doses of 5-FU, HCQ, or CC1-779 as indicated in the Figure, during 48 h. The cell viability was examined by the MTT assay at the end of the incubation period, and normalized with respect to that obtained in untreated cells (Ctr). The bar graph shows the means ± SEM from at least three independent experiments.

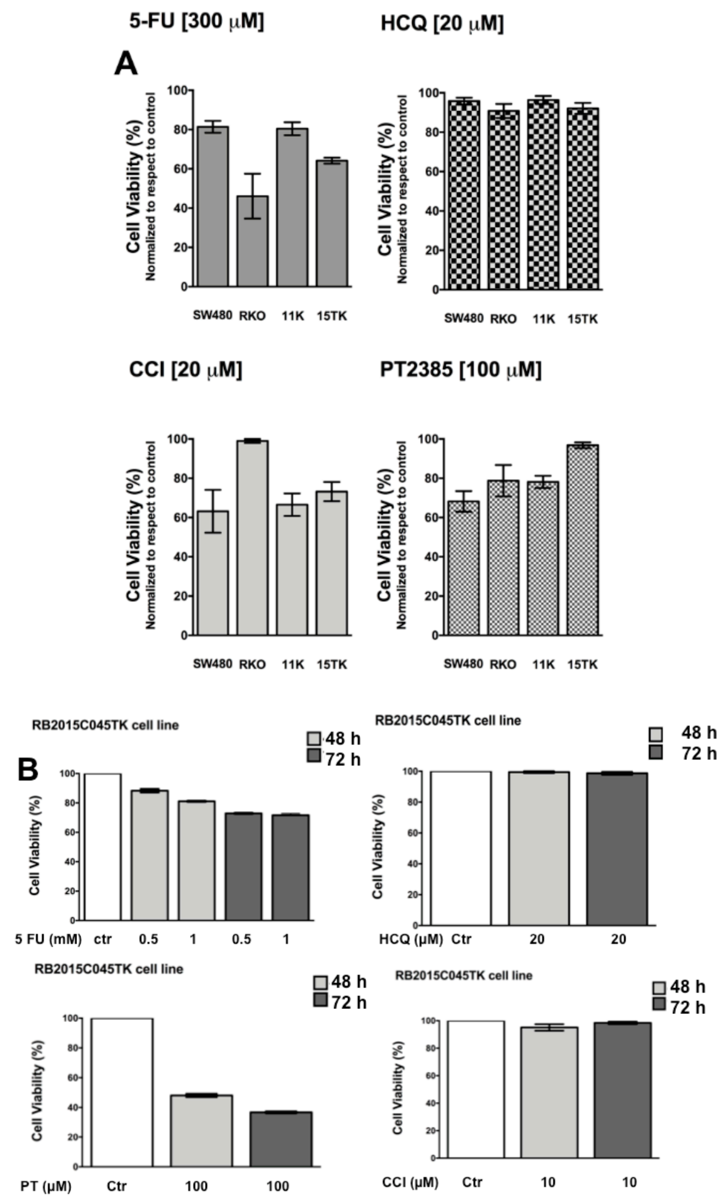

Figure S5. 19739-11K cells and OMCR14-015TK cells displayed resistance against 5-FU treatment (**A**), whereas OMCR15-045TK (**B**) displayed resistance to both 5-FU and CCI-779. (**A**) Patient-derived 19739-11K and OMCR0-015TK cells or SW480 and RKO cell lines were incubated in the presence of the indicated concentrations of 5-FU, HCQ, CCI-779 or PT-2385 during 48 h or 72 h as indicated in the figure. The cell viability was examined by the MTT assay at the end of the incubation period. The bar graph shows the means ± SEM from at least three independent experiments. (**B**) Patient-derived OMCR15-045TK cells were incubated in the absence (Ctr) or the presence of the indicated concentrations of 5-FU, HCQ, PT-2385 or CCI-779 during 48 h or 72 h. The cell viability was examined by the MTT assay at the end of each incubation period. The bar graph shows the means ± SEM from at least three independent experiments.

© 2019 by the authors. Licensee MDPI, Basel, Switzerland. This article is an open access article distributed under the terms and conditions of the Creative Commons Attribution (CC BY) license (http://creativecommons.org/licenses/by/4.0/).
